# Inclusiones azul-verdosas en los neutrófilos acompañadas de fallo hepático como señal de muerte inminente

**DOI:** 10.1515/almed-2022-0001

**Published:** 2022-07-11

**Authors:** Antonio Sierra Rivera, María García Valdelvira, María Asunción Elia Martínez, Ángeles Férez Martí, Goitzane Marcaida Benito

**Affiliations:** Servicio de Análisis Clínicos Consorcio Hospital General Universitario de Valencia, Valencia, España; Servicio de Análisis Clínicos Hospital Universitario Doctor Peset, Valencia, España

**Keywords:** acidosis láctica, cristales verdes de la muerte, fallo hepático severo

## Abstract

**Objetivos:**

Las inclusiones azul-verdosas, birrefringentes y de contornos poco definidos que se encuentra en el citoplasma de algunos leucocitos son un hallazgo infradiagnosticado y han sido motivo de discusión por parte de los profesionales en cuanto a su composición y repercusión clínica. La extensión de sangre periférica (ESP) es el único procedimiento que permite la detección de estas inclusiones.

**Caso clínico:**

Se presenta el caso correspondiente an un varón con antecedentes de enfermedades crónicas (hipertensión arterial, obesidad, dislipemia, insuficiencia renal) atendido inicialmente en el Servicio de Urgencias por disnea y que requirió de ingreso en la unidad de cuidados intensivos (UCI). Su curso clínico evolucionó negativamente derivando en su fallecimiento por fallo multiorgánico, incluyendo el fallo hepático severo. En el momento del empeoramiento observamos estas inclusiones en la ESP simultáneamente a la alteración de los parámetros de función hepática.

**Conclusiones:**

Confirmamos que la detección de estas inclusiones mediante ESP, en casos de fallo hepático severo agudo acompañado de acidosis láctica, es relevante notificarla lo antes posible al clínico para alertar de la situación crítica del paciente, dada su asociación con un mal pronóstico.

## Introducción

Las inclusiones de color azul-verdoso en el citoplasma de los leucocitos son un raro hallazgo que suele ir asociado a una elevada mortalidad. Estas inclusiones aparecen principalmente en los neutrófilos, aunque también pueden aparecer en los monocitos en un menor porcentaje. Hasta el momento, se han publicado escasos artículos que incluyen alrededor de 70 pacientes en los que se han podido encontrar este tipo de inclusiones [1]. La presencia de estos depósitos citoplasmáticos parece ser debida a sustancias como la biliverdina, la lipofuscina o similares, aunque su origen no está claramente definido. Este tipo de inclusiones se han denominado “cristales verdes de la muerte” debido a que la gran mayoría de los pacientes en los que han sido descubiertos fallecen en un corto periodo de tiempo, entre las 24–48 h siguientes.

Los pacientes que permanecen un largo periodo en Unidad de cuidados intensivos (UCI) son susceptibles de desarrollar un fallo multiorgánico en el que puede ir incluido un fallo hepático agudo que determine la aparición de estas inclusiones [2]. Además, se ha observado que el riesgo de mortalidad se incrementa cuando hay fallo hepático asociado a acidosis láctica, ya que la aparición de acidosis es un predictor negativo de supervivencia [3, 4].

A continuación, describimos un caso de inclusiones azul-verdosas asociado an un fallo hepático ocurrido en nuestro hospital.

## Caso clínico

Paciente varón de 66 años caucásico que acudió al servicio de urgencias de nuestro hospital con síntomas de disnea. Entre sus antecedentes destacó: hipertensión arterial, dislipemia, obesidad e insuficiencia renal en estadio IV.

Debido a la baja saturación de oxígeno que presentó en el momento de su llegada, el paciente ingresó en UCI con oxigenoterapia y monitorización. En ese momento, el médico responsable del paciente le solicitó una analítica completa con perfil hematológico, hemostasia y bioquímica, radiografía de tórax, reverse transcription-polymerase chain reaction (RT-PCR) de SARS-CoV-2 y detección de diversos virus respiratorios.

El resultado de la RT-PCR de SARS-CoV-2 (GeneXpert, Cepheid^®^) fue positiva. Entre los resultados analíticos destacaron unos niveles elevados de creatinina (7,04 mg/dL) y de proteína C reactiva (26 mg/dL) junto a otros parámetros también alterados a causa de la infección por COVID-19, los cuales quedan reflejados en la Tabla 1. La radiografía de tórax mostró una neumonía bilateral con infiltrados alveolares periféricos bilaterales. El estudio por PCR del resto de virus respiratorios como virus respiratorio sincitial (VRS), rinovirus y adenovirus (PCR multiplex FilmArray, Biomerieux^®^) fue negativo.

**Tabla 1:** Evolución de los parámetros hematológicos y bioquímicos el día de ingreso, el día previo a la aparición de las inclusiones y el día de la detección de las mismas.

HEM/BQ	Ingreso	Día 24 (8 am)	Día 24 (12 pm)	Día 25 (8 am)	Unidades	Intervalos de referencia
Leucocitos	9,9	19	48,7	65,2	10^9^/L	3,8–10,8
Neutrófilos	8,8	15,5	39,7	43,7	10^9^/L	1,8–7,5
Linfocitos	0,6	1,3	4,6	3,3	10^9^/L	1,5–4
Monocitos	0,4	1,3	3,5	5,9	10^9^/L	0,2–0,8
Hemoglobina	12,3	8,9	7,9	11,3	g/dL	13,5–18
Plaquetas	178	233	356	252	10^9^/L	135–350
Frotis SP			No se refleja la existencia de inclusiones en los neutrófilos	Se observan inclusiones citoplasmáticas en los neutrófilos. Anisocitosis plaquetar y eritrocitaria		
ALT/GPT	28	23	25	1284	U/L	10–45
AST/GOT	–	20	33	1401	U/L	5–35
GGT	–	303	245	319	U/L	8–55
Bb total	0,5	0,52	0,51	1,15	mg/dL	0,3–1,2
LDH	1288	650	813	6023	U/L	208–378
FA	54	142	111	131	U/L	30–120
Lactato	1,2	0,5	2,2	10,6	mmol/L	0,5–2
Creatinina	7,04	3,93	3,12	2,59	mg/dL	0,67–1,17
Urea	178	245	169	136	mg/dL	17–43
Prot C reac	25,9	12,3	10,9	13	mg/dL	0,00–0,5

HEM, hemograma; BQ, bioquímica; SP, sangre periférica; ALT, alanina aminotransferasa; GPT, glutamato-piruvato transaminasa; AST, aspartato aminotransferasa; GOT, glutamato-oxalacetato transaminasa; GGT, gamma glutamiltransferasa; Bb, bilirrubina; LDH, lactato deshidrogenasa; FA, fosfatasa alcalina; Prot C reac, proteína C reactiva.

A la semana del ingreso, la enfermedad renal en estadio IV evolucionó a estadio V, por lo que se decidió iniciar técnicas de reemplazo renal continuo (TRRC). Durante su estancia en UCI, el paciente presentó hemocultivos positivos para *Candida auris* y *Staphylococcus aureus,* para los cuales se pautó tratamiento con Isavuconazol y Daptomicina. Así mismo, el cultivo de esputo mostró crecimiento de *Pseudomonas aeruginosa* y *Stenotrophomonas maltophilia* pautándose también Piperacilina/Tazobactam y Tobramicina. En la tercera semana en UCI, el paciente sufrió una parada cardiorrespiratoria de la que fue reanimado. En el tomografía axial computarizada (TAC) posterior a la parada se pudo apreciar un derrame tanto pericárdico como pleural. La inestabilidad hemodinámica del paciente hizo que tuviera que recibir hasta 22 transfusiones durante el periodo de ingreso.

El día 25 de su estancia hospitalaria, en la analítica de control diaria, el hemograma (DxH 800 de Beckman Coulter^®^) mostró leucocitosis con desviación izquierda, monocitosis, alarma de blastos en área mieloide, hemoglobina de 11,3 g/dL y recuento plaquetar de 252 × 109/L por lo que se procedió a realizar un frotis de sangre periférica mediante la tinción de May-Grünwald Giemsa.

**Figura 1:**
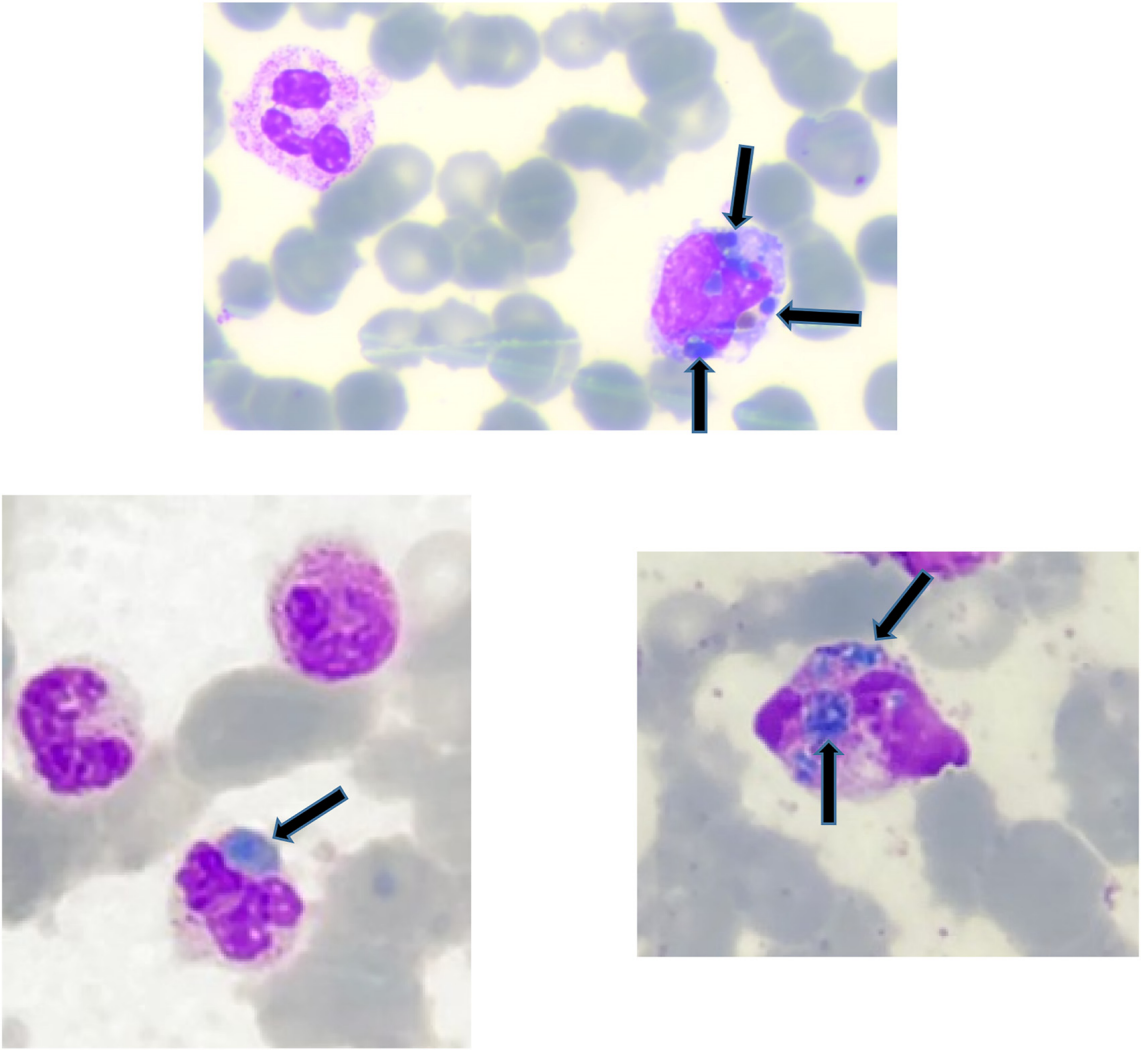
Inclusiones citoplasmáticas azul-verdosas en frotis de sangre periférica (May-Gründwald Giemsa).

En la extensión de sangre periférica (ESP) se confirmó la desviación izquierda (67% segmentados, 11% cayados, 1% metamielocitos y 6% mielocitos), observándose en el citoplasma del 1–2% de los neutrófilos y en algún monocito la presencia de inclusiones azul-verdosas, de contornos poco definidos, con refringencia (Figura 1).

En los datos bioquímicos el paciente presentó una elevación en los valores de alanina aminotransferasa (ALT) y de lactato, por lo que desde el laboratorio se decidió ampliar el resto de pruebas del perfil hepático como la aspartato aminotransferasa (AST), gamma glutamiltransferasa (GGT), bilirrubina (Bb) y fosfatasa alcalina (FA), además de la lactato deshidrogenasa (LDH). Los resultados obtenidos se muestran en la Tabla 1. En ella se muestra el evolutivo de los parámetros bioquímicos y hematológicos del paciente al ingreso, el día previo a la aparición de las inclusiones y el día de la detección de las mismas.

Además, en la Tabla 1 podemos apreciar un incremento muy significativo de las concentraciones de ALT, AST y de LDH en el momento del fallo hepático. Si nos fijamos en los valores bioquímicos podemos comprobar que el día anterior los parámetros estaban dentro de los intervalos de referencia o ligeramente aumentados, hasta el momento en el que se desencadenó el fallo hepático en el que aumentaron considerablemente la gran mayoría de ellos.

Tras la visualización de los cristales en el frotis de sangre periférica, nuestro servicio comunicó de manera inmediata este hallazgo al médico responsable del paciente. A pesar de ello, el paciente falleció unas horas más tarde a causa de un fallo multiorgánico.

## Discusión

El origen exacto de estas inclusiones azul-verdosas, su morfología y su composición están aún por determinar [5, 6]. Hay diversas hipótesis, en el contexto del fallo hepático agudo se ha sugerido que estas inclusiones pueden deberse a productos biliares que circulan por sangre o a productos de degradación lisosomal fagocitados por neutrófilos y monocitos. Un ejemplo es el caso del pigmento verde conocido como biliverdina. Otros autores creen que puede ser debido an una sustancia similar a la lipofuscina, que es liberada por las células necróticas del parénquima hepático [3, 6], [7], [8].

Es importante reconocer e investigar este tipo de inclusiones y su significado clínico, así como diferenciarlas de otros tipos de inclusiones también presentes en el citoplasma de neutrófilos como pueden ser los cuerpos de Döhle, pseudocuerpos de Howell–Jolly, crioglobulinas, anomalía de May-Hegglin, etc.

El significado clínico es incierto aunque se ha encontrado una fuerte asociación con pacientes que sufren un fallo hepático agudo y severo. Se ha reportado un alto índice de mortalidad en un corto periodo de tiempo en pacientes que presentan estas inclusiones, pero está limitado an unos pocos casos descritos en la literatura [7].

Otros autores han descrito que al principio de la enfermedad por SARS-CoV-2 el 80% de los pacientes presentan linfocitopenia, la cual se normaliza después de la detección de las inclusiones. Si nos fijamos en la Tabla 1 podemos comprobar como este caso refuerza esta teoría [9].

La revisión del frotis de sangre periférica es una prueba fundamental que complementa la información proporcionada por el hemograma automatizado ya que existen determinados aspectos morfológicos, como estos cristales, que no pueden ser evaluados mediante los analizadores hematológicos. De aquí que la revisión microscópica por parte del profesional adquiera un valor añadido a la hora de orientar sobre la situación clínica del paciente y sobre posibles pruebas complementarias a realizar, como la ampliación del perfil hepático y LDH en este caso, para un mayor conocimiento de la patología, evolución y pronóstico de la enfermedad.

Podría ser de interés la realización de futuros estudios para revelar la composición de estos cristales. Así como dar a conocer todos los posibles casos en los que sean observados junto a sus respectivas imágenes, para así facilitar su reconocimiento en la ESP por parte de los profesionales del laboratorio clínico.

Nuestro caso respalda la literatura actual publicada y sirve como recordatorio de que la presencia de inclusiones azul-verdosas en el frotis periférico, junto con fallo hepático acompañado de acidosis láctica, puede usarse como predictor de mortalidad en pacientes críticamente enfermos con insuficiencia hepática [4, 10]. La información que proporciona el laboratorio es fundamental para el pronóstico de los pacientes.

## Puntos clave de aprendizaje

– Las inclusiones azul-verdosas conocidas como “cristales verdes de la muerte” son un hallazgo infradiagnosticado.
– La rapidez de actuación del laboratorio resulta esencial a la hora de notificar la aparición de estas inclusiones para alertar de la situación crítica que presenta el paciente.
– Estas inclusiones son debidas an un fallo hepático agudo asociado a acidosis láctica.
– Importancia de la realización de ESP como método de detección de estas inclusiones.
